# Synchronization between music dynamics and heart rhythm is modulated by the musician’s emotional involvement: A single case study

**DOI:** 10.3389/fpsyg.2022.908488

**Published:** 2022-09-08

**Authors:** Laura Sebastiani, Francesca Mastorci, Massimo Magrini, Paolo Paradisi, Alessandro Pingitore

**Affiliations:** ^1^Department of Translational Research and New Technologies in Medicine and Surgery, University of Pisa, Pisa, Italy; ^2^Institute of Information Science and Technologies “Alessandro Faedo”, National Research Council (ISTI-CNR), Pisa, Italy; ^3^Institute of Clinical Physiology, National Research Council (IFC-CNR), Pisa, Italy; ^4^Basque Center for Applied Mathematics (BCAM), Bilbao, Spain

**Keywords:** musician, live performance, music-heart synchronization, emotions, technical demands

## Abstract

In this study we evaluated heart rate variability (HRV) changes in a pianist, playing in a laboratory, to investigate whether HRV changes are guided by music temporal features or by technical difficulty and/or subjective factors (e.g., experienced effort). The pianist was equipped with a wearable telemetry device for ECG recording during the execution of 4 classical and 5 jazz pieces. From ECG we derived the RR intervals series (tachogram), and, for each piece, analyzed HRV in the time (RR, RMSSD, Stress Index) and frequency domains (Total spectral power) and performed non-linear analysis (Multiscale Entropy). We also studied the correlation (Pearson) between the time course of music volume envelope and tachogram. Results showed a general reduction of parasympathetic and an increase of sympathetic activity, with the greatest changes during the classical pieces execution, the pianist appraised as more demanding than the jazz ones. The most marked changes occurred during the most technically/emotionally demanding piece, and correlation analysis revealed a negative association between music volume envelope time course and tachogram only for this piece, suggesting a modulation of the limbic system on the synchronization between heart rhythm and music temporal features. Classical music was also associated with the increase of entropy (1st scale) with respect to rest, indicating its effectiveness in driving flexible, healthy, heart dynamics. In conclusion, HRV seems modulated not only by the music temporal features, but also by the pianist’s emotional involvement, which is greatly influenced, in a non-trivial manner, by the technical demands and musician expertise.

## Introduction

A musical performance in front of an audience is a highly challenging situation and, thus, performance success or failure depends on the ability of the musician to manage physical, cognitive and emotional demands. The psychophysical effort is typically accompanied by autonomic changes, such as increased heart rate and blood pressure and a decreased heart rate variability (HRV) that are sustained by an increased sympathetic activation (Williamon et al., 2013).

In a previous study (Sebastiani et al., 2020) we evaluated changes in heart autonomic modulation in a skilled pianist, during the execution of a live concert. Throughout the whole musical session, it was observed a general trend, in particular, a decrease of HRV parasympathetic indices and an increase of the sympathetic ones. Nonetheless for each musical piece, the dynamics of the autonomic modulation were very different. This piece-specific modulation could be accounted for by the different effort required to play pieces with different technical difficulty and/or by the peculiar expressive/emotional features of each performance. Interestingly, we also found a negative correlation between the temporal dynamic of music volume envelope that is a measure of the time course of sound intensity, and the series of beat-to-beat RR intervals, which could be interpreted as the expression of music-heart rhythm synchronization (Sebastiani et al., 2020).

In the present single-case study, carried out in a laboratory setting, we evaluated HRV changes in a pianist while playing, before a small audience, two different types of music, namely classical and jazz, characterized by different levels of technical difficulty. The pianist was not a professional musician and possible motor learning effects were unlikely since the pianist knew the played musical pieces but no in-depth training of them had preceded the live performance. The rationale of the present study was that music features and also music technical difficulties could interfere with the emotional status of the pianist while playing music. Thus, we explored whether HRV changes are guided by specific music features or rather by music technical difficulty and/or subjective factors (e.g., experienced effort).

## Materials and methods

### Participant

The music player was a young healthy man of 35 age. He was not a professional musician, but he had studied classical piano and regularly played jazz in public as well. The experiment consisted of a single music session performed in a laboratory setting with the experimenters and a few more people as the audience, for a total of nine persons. This study was approved by the Committee on Bioethics of the University of Pisa (Review No. 15/2021) and the participant signed the informed consent to participate in the study.

### Experimental protocol

The pianist played 9 pieces with a MIDI keyboard connected to a computer. The interval among the musical pieces was 2 min. Four pieces were excerpts from the “Das Wohltemperierte Klavier, I, of J.S. Bach, and the other 5 were jazz pieces (see Supplementary Table 1). The music player practiced both classical and jazz pieces in the 4 weeks proceeding the experimental session for a total of about 16 h.

At the end of the performance the pianist was asked to score the technical difficulty of each piece as well as the subjective, experienced difficulty in playing each piece in a 5-points Likert scale (1 = very easy, 5 = very difficult). The music player was equipped with the wearable telemetry device (Bioharness 3 Zephyr) for recording of ECG (sampling rate 250 Hz) during a relaxation period (5 min) preceding the performance and during the execution of the 9 music pieces.

### Heart rate variability analysis

We analyzed ECG by means of Kubios HRV Premiums software, which is nowadays a standard validated tool that allows the ECG analysis from the artifact removal to the evaluation of time, frequency and non-linear indices^1^. The signals were controlled for artifacts and ectopic beats were removed by means of a threshold-based correction as well as through a validated automatic artifact correction method (Lipponen and Tarvainen, 2019). When necessary, beat detections were corrected manually. We derived the beat-to-beat RR intervals, thus obtaining the RR time series (tachogram), and carried on the analysis of HRV in time [mean RR, root mean square of successive (RR) differences (RMSSD), Stress Index (SI)] and frequency domains (Total spectral power, TP). This was done in order to evaluate the autonomic changes associated with the different musical pieces. RMSSD is an index of parasympathetic activity within the autonomic regulation and is calculated as the square root from the sum of squared differences of sequential RR intervals. RMSSD normal values are usually within the range of 20–50 ms and the higher RMSSD, the more active parasympathetic regulation. The evaluation of SI is based on the histogram of RR interval distribution. Specifically, it is computed as the square root of the ratio of histogram height to width (Baevsky and Chernikova, 2017) and its increment reflects sympathetic tone rise.

TP is a standard measure of HRV in the frequency domain (see text footnote 1) and is evaluated by means of the square of the Fast Fourier Transform (FFT) applied to RR signal. Following standard HRV frequency analysis, we estimated the TP in the 0.003–0.4 Hz band. TP is highly correlated with the square root of the standard deviation of the RR intervals (SDNN) and has been interpreted as a marker of stress (Baevsky and Chernikova, 2017; Shaffer and Ginsberg, 2017).

We also carried out non-linear analysis of the tachogram. In fact, previous studies indicated that tachograms fluctuate in a complex way, and suggested that non-linear methods, such as sample entropy (SE) are able to describe changes in HRV that cannot be caught by linear methods (Shekatkar et al., 2017; Solís-Montufar et al., 2020). In short, SE applied to HRV provides information about the predictability of the fluctuations in successive RR intervals, which reflects the complexity of HRV regulatory mechanisms: large values indicate low predictability of fluctuations and/or high randomness, while small values indicate high predictability and regularity (smoothness) of the RR series. In the present study we applied Multiscale Entropy (MSE), which is an extension of Sample entropy (SE) to multiple time scales of measurement and allows to quantify the degree of predictability and irregularity over a range of coarse-grained scales (Costa et al., 2002, 2005).

### Analysis of the correlation between music envelope time course and tachogram

In order to investigate the possible association between specific music temporal features and heart rate we derived from the MIDI recording of the performance the time course of the music volume envelope of each piece, which is a measure of the temporal dynamics of music loudness. We measured music volume envelope by means of the Root Mean Square (RMS) which allows calculating the average of audio signals over a period of time. The signal amplitude is squared, averaged over a period of time (0.5 s), then the square root of the result is calculated. The resulting value is proportional to the effective power of the signal.


$${\text{X}}_{R\,M\,S}=\sqrt{\frac{1}{N}\,\sum_{1}^{N}{\left|X_{n}\right|}^{2}}$$

*Sampling Rate = 44,100*

*Segments duration = 0.5 s*

N = 0.5*44,100 = 22,050

We then studied the correlation (Pearson Correlation analysis) between RMS and the RR time series recorded during each piece. We set the correlation coefficient cutoff at ± 0.3. We analyzed the statistical significance of the correlation by means of Student’s *t*-test. Significance was set at *p* < 0.05.

## Results

Following the structured interview, the pianist evaluated the classical pieces as technically more difficult than the jazz ones (see Supplementary Table 1). Also, he reported a subjective greater difficulty in playing two classical pieces, namely the 2nd scored 4 and the 4th scored 5, with respect to all the other pieces (score 3) (see Supplementary Table 1). Besides, the pianist reported a very strong emotional involvement (“I felt under pressure”; “I was afraid to make mistakes”) while playing the 4th classical piece, which he defined as “his cursed piece.”

**FIGURE 1:**
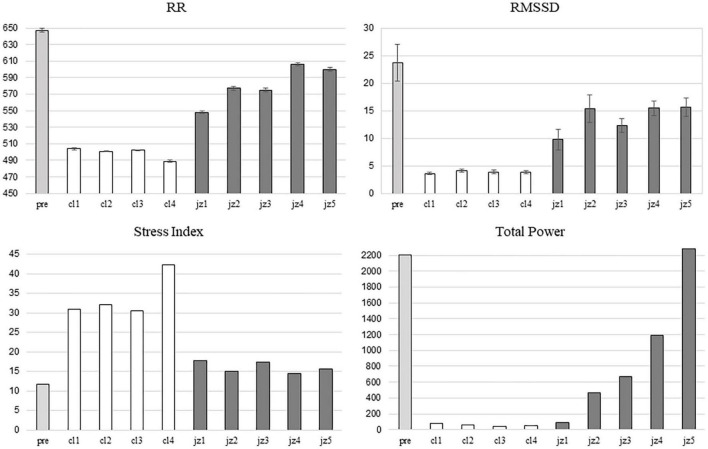
Mean value of RR, RMSSD, Stress Index and Total power recorded in the pianist during the basal period (pre, light grey bars), the 4 classical pieces (cl1-cl4, white bars) and the 5 jazz pieces (jz1-jz5, grey bars) are shown.

Figure 1 shows the mean value of RR, RMSSD, Stress Index and Total power recorded in the pianist during the basal period and each of the 9 pieces.

As can be observed a decrease of mean RR and of RMSSD was found in the music session with respect to the basal period. However, the reduction in mean RR and RMSSD was greater during the classical music sub-session than in the jazz one. An increase of SI was also found during the music session with respect to the basal period, with higher values during the classical music sub-session than the jazz one. The highest level of SI occurred during the performance of the 4th classical piece (3.6 times greater than the basal value).

During the music session, a remarkable reduction of TP, that was more pronounced during the classical music sub-session, was observed (Figure 1). Indeed, TP recovery occurred during the jazz session in which TP mean value was similar to the basal one in the last jazz piece.

**FIGURE 2:**
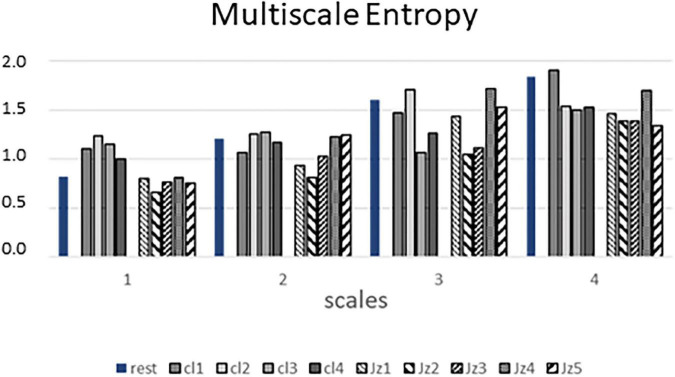
Figure shows the results of the Multi Sample Entropy analysis for the first 4 scale factors relative to the basal period (rest, blue bars), the 4 classical pieces (cl1-cl4, grey bars) and the 5 jazz pieces (jz1-jz5, striped bars).

Figure 2 shows the results of the MSE analysis for the first 4 scale factors. In general, for both the rest condition and all the musical pieces, MSE values tended to increase for higher scale factors. The result indicated higher SE for the classical pieces than for the jazz ones when the first scale is considered.

**FIGURE 3:**
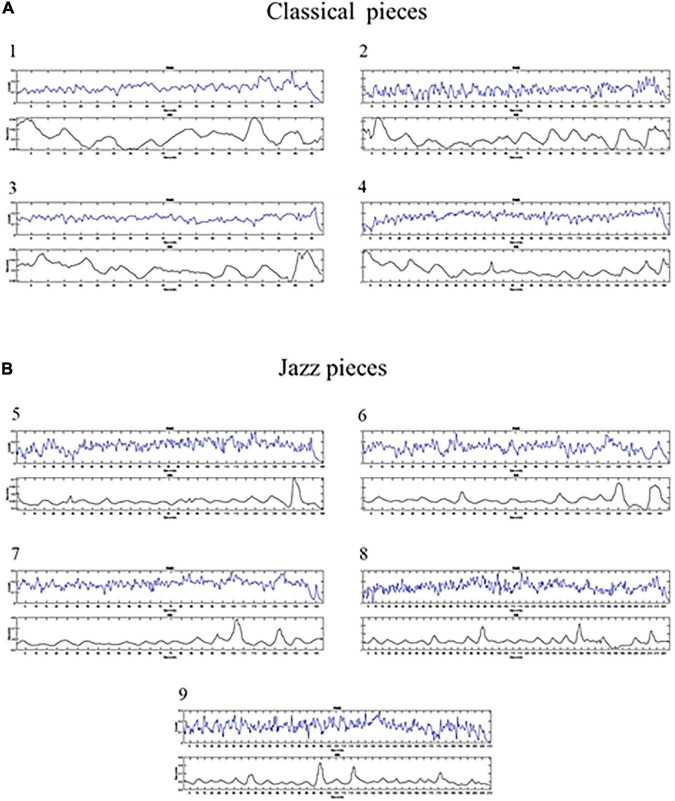
Figure shows the time course of the music volume envelope (upper panel) and the RR time series (lower panel) of the 9 pieces [1–4 classical pieces, **(A)**; 5–9 jazz pieces, **(B)**].

Pearson correlation analysis between the time course of the music volume envelope and the RR time series recorded in the pianist during the execution of each piece revealed a moderate negative linear association (*r* = −0.46125, *p* < 1.5848 e^–14^) only for the 4th classical piece. In Table 1 the correlation coefficients (r) relative to all the music pieces are reported, while in Figure 3 the time course of the music volume envelope (upper panel) and the RR time series (lower panel) of the 4 classical (Figure 3A) and the 5 jazz pieces (Figure 3B) are shown.

**TABLE 1:** Correlation analysis between music envelope and RR.

Music	Piece *n*°	*r*	*P*
Classic	1	0.024419	0.74076
	2	0.052422	0.38564
	3	0.20727	0.0045305
	4	**−0.46125**	**1.5848 e^–14^**
Jazz	5	0.14438	0.0097035
	6	0.14823	0.010271
	7	0.26408	4.10*E*−02
	8	0.085211	0.071574
	9	0.032208	0.50986

Significative values are indicated in bold.

## Discussion

The present study explores HRV changes in a pianist while playing in a laboratory setting in order to investigate whether these changes are guided by specific music features or rather by music technical difficulty and/or subjective factors (e.g., experienced effort). The emotions experienced by the pianist were not directly assessed by means of specific tests, but only through the analysis of HRV, which is an indirect expression of affective dimension, perceived anxiety and stress (Kim et al., 2018).

The results of this single-case study showed a general reduction of parasympathetic activity associated with an increase of the sympathetic activation in the pianist during the musical performance with greater changes during the execution of the classical pieces than the jazz ones.

Specifically, RR were lower than the basal values across the whole music session with the lowest values during the classical sub-session and a gradual increase toward basal values from the 1st to the 5th jazz piece. A similar pattern was observable for RMSSD whose values dropped below 4 during the classical music performance, thus indicating a strong parasympathetic withdrawal.

These findings are in accord with those of Harmat and Theorell (2010), which found increased HR and suppressed HRV during high-stress musical performances than in low-stress ones.

Furthermore, the sharp reduction of TP during classical music performance is in line with a strong inhibition of parasympathetic control. TP provides the range of HRV around its mean value and is highly correlated with SDNN (Baevsky and Chernikova, 2017; Shaffer and Ginsberg, 2017).

The reduction of both measures suggests the activation of sympathetic regulation that inhibits the autonomic parasympathetic, mainly respiratory, regulation of heart rate. TP and SDNN have been found to decrease in stressful conditions and, in general, low values of these measures have been associated with negative feelings and lowered ability to cope with emotional/physical stress (Baevsky and Chernikova, 2017; Shaffer and Ginsberg, 2017; Kim et al., 2018).

Changes in this parasympathetic index are associated with a strong increase of SI, which characterizes the activity of sympathetic regulation (Baevsky and Chernikova, 2017). Activation of sympathetic regulation during mental/physical stresses manifests itself with heart rate stabilization, decrease of the range of RR intervals duration, and increase of the number of RR intervals with similar duration. In the histogram of RR interval distribution, it corresponds to the narrowing of the distribution and growth in height that is an increment of SI. SI, in fact, is very sensitive to sympathetic tone rise: a mild physical/emotional stress produces a 1.5–2-fold increase in the index, while intense stress a 5–10-fold increase (Baevsky and Chernikova, 2017).

In line with these previous data, our results show that SI reaches a peak value during the 4th classical piece, which, indeed, is considered by the player the most technically difficult and the most demanding from a personal point of view.

These results are in accordance with what we found in our previous study on the virtuoso pianist during a live concert (Sebastiani et al., 2020). In fact, also in that case a general reduction of parasympathetic variability and an increase of sympathetic indices throughout the whole musical session was observed.

The pattern of the observed autonomic changes could reflect music performance anxiety which is known to be associated with different factors including the difficulty of the performance (Kenny, 2011), the performance environment and the importance attributed by the musician to his performance (Le Blanc et al., 1997; Spahn et al., 2021). Thus, we cannot exclude that also the lab experimental context may have contributed to increase the musician anxiety being the whole experimental session centered on his performance.

Results of non-linear analysis based on the measure of MSE showed that the execution of the classical pieces, which was associated with low HRV, yielded an increase of Sample Entropy (1st scale factor of MSE) with respect to both the rest condition and the jazz session. This observation confirms that variability and complexity indices do not necessarily describe the same features of dynamical signals- e.g., a sinusoidal signal has high variability but low entropy- and that classical music could be particularly effective in driving flexible, thus healthy, heart rate dynamics.

However, the difference in the 1st scale factor of MSE between classical music and both rest condition and jazz music was not maintained in the other scales. In fact, a general increase of MSE from the 1st to the 4th scale occurred in all the conditions (see Figure 2).

Entropy of a dynamical system is a measure of its complexity that refers to information contained in its actual state: increased values indicate a more complex signal while decreased values less complexity. Time-series with high degree of regularity have the lowest entropy values, while signals fluctuating more freely have high entropy values (Xiong et al., 2017). Complexity is recognized as an intrinsic property of healthy biological systems, and the loss of complexity, for example with aging and disease, has been interpreted as a marker of reduced adaptive capabilities of the individual. Also, studies on HRV complexity based on entropy approaches, either Approximate, Sample or Multiscale, suggested that complexity is lowest during states of high stress (Buchman and Karsch, 2009; Turianikova et al., 2011; Williamon et al., 2013; Solís-Montufar et al., 2020).

Present findings on MSE contrast with the results we previously obtained in the skilled pianist’s performance. In that case, throughout the whole musical session, we found a substantial decrease of Approximate Entropy with respect to the pre-performance rest condition, which was consistent with previous evidence of lowest complexity in states of high stress (Williamon et al., 2013).

Interestingly, in a recent study Solís-Montufar et al. (2020) associated changes in HRV entropy to fatigue. In particular, the authors described an increase of HRV entropy in trained persons during moderate physical activity and a decrease when the physical activity lasted for a long period, that is when fatigue accumulates. Similarly, our results showed that SE values increased in the first part of the performance, began to decrease starting from the execution of the highly demanding piece (the 4th classical piece) and remained a little below, even if close, to the rest values during the second part of the musical session. Thus, we cannot exclude that this decrease in entropy could reflect both physical and mental fatigue. On the other hand, the whole musical performance of the virtuoso pianist was highly demanding from both a technical and expressive/emotional point of view, which could justify the marked decrease of entropy from the very beginning of the performance.

Analysis of correlation between the temporal dynamics of music volume envelope and the RR series revealed a significant negative association between them only for the 4th classical piece. As previously underlined, this piece was very difficult, and it also evoked in the performer intense negative feelings. The match between heart rhythm and this temporal feature of music could, thus, reflect the pianist’s emotional response to music, and we may assume that this association could be the expression of the entrainment between emotionally meaningful music and activity in brain areas involved in the physiological expression of emotion (Juslin et al., 2014). Previous studies showed that specific acoustic features of music are linked with particular emotional responses (Gabrielson and Juslin, 2003; Schubert, 2004) and are able to differentially modulate the activity of neural regions involved in emotional/cognitive processing (Trost et al., 2015, 2017; Wollman et al., 2020). For instance, energy-related musical features have been found to be negatively correlated with the activity in the limbic system (i.e., left amygdala and nucleus accumbens) (Trost et al., 2015).

## Conclusion

This preliminary study, which was carried out on a single musician, gave interesting results about the interplay between music features and HRV of the musician. In summary, HRV seems mostly modulated by classical music, which, likely owing to its temporal (e.g., sound intensity fluctuation over time) but also non-temporal (e.g., harmony) features, appears to be particularly able to entrain activity in emotional-related brain regions that control heart rhythm. However, the technical demands, the musician’s expertise as well as the environment are all factors that affect a pianist’s subjective emotional involvement, likely shaping (i.e., increasing or decreasing) heart-music synchronization.

In our opinion, the present results, which cannot be generalized in the present form, could provide hints for developing experimental protocols in future investigations extending the statistical sample to a group of musicians. Further studies are needed, not only to include more pianists, but also to assess, through specific tests and questionnaires, other aspects that may modulate the complex dynamical interplay between music and heart rhythm, such as personality properties, personal stress resistance, anxiety, affective lability, and fatigue.

## Data availability statement

The raw data supporting the conclusions of this article will be made available by the authors, without undue reservation.

## Ethics statement

The study involving human participants was reviewed and approved by the Committee on Bioethics, University of Pisa (Review No. 15/2021). The participant provided his written informed consent to participate in this study.

## Author contributions

LS and PP developed the main idea of the manuscript. All authors participated in the development of the experimental protocol and carried out the experimental session. LS and MM analyzed the recorded variables and performed the statistical analysis. LS drafted the manuscript. All authors revised and edited the manuscript, read and approved the final version of the manuscript, and agreed with the order of presentation of the authors.

## Footnotes

1
https://www.kubios.com/hrv-analysis-methods/
