# Supplementary material for: Synchronization between music dynamics and heart rhythm is modulated by the musician’s emotional involvement: A single case study
Source: Front Psychol. 2022 Sep 8;13:908488. doi: 10.3389/fpsyg.2022.908488 (PMC9493261; doi:10.3389/fpsyg.2022.908488)
Supplement: Supplementary file 1 [file Table_1.DOCX]

| Type | Piece and author | | | | | technical | experienced |
| --- | --- | --- | --- | --- | --- | --- | --- |
|  |  |  |  |  |  | difficulty | difficulty |
| Classic 1 | Prelude n.5, Das Wohltemperierte Klavier, I, J.S. Bach | | | | | 3 | 3 |
| Classic 2 | Fugue n.5, Das Wohltemperierte Klavier, I, J.S. Bach | | | | | 3 | 4 |
| Classic 3 | Prelude n.3, Das Wohltemperierte Klavier, I, J.S. Bach | | | | | 3 | 3 |
| Classic 4 | Fugue n.3, Das Wohltemperierte Klavier, I, J.S. Bach | | | | | 4 | 5 |
| Jazz 1 | There will never be another you, H. Warren | | | |  | 2 | 3 |
| Jazz 2 | Everything happens to me, M. Dennis | | | |  | 2 | 3 |
| Jazz 3 | Someday my prince will come, F. Churchill | | | |  | 2 | 3 |
| Jazz 4 | Mood Indigo, D. Ellington | | |  |  | 2 | 3 |
| Jazz 5 | Prelude to a kiss, D. Ellington | | |  |  | 2 | 3 |

Table 1. Played pieces: technical and experienced difficulty

Note: scores are given in a 5-points Likert scale
